# Serum alanine aminotransferase to hemoglobin ratio and radiological features predict the prognosis of postoperative adjuvant TACE in patients with hepatocellular carcinoma

**DOI:** 10.3389/fonc.2022.989316

**Published:** 2022-09-16

**Authors:** Zicong Xia, Yulou Zhao, Hui Zhao, Jing Zhang, Cheng Liu, Wenwu Lu, Lele Wang, Kang Chen, Junkai Yang, Jiahong Zhu, Wenjing Zhao, Aiguo Shen

**Affiliations:** ^1^ Cancer Research Center Nantong, Tumor Hospital Affiliated to Nantong University, Medical School of Nantong University, Nantong, China; ^2^ Department of Interventional Radiology, Afiliated Hospital of Nantong University, Nantong, China

**Keywords:** postoperative adjuvant transarterial chemoembolization, Alanine aminotransferase to hemoglobin ratio, prognosis, nomogram, peritumor capsule, rim-like arterial phase enhancement, hepatocellular carcinoma

## Abstract

**Objective:**

To explore the prognostic value of radiological features and serum indicators in patients treated with postoperative adjuvant transarterial chemoembolization (PA-TACE) and develop a prognostic model to predict the overall survival (OS) of patients with hepatocellular carcinoma (HCC) treated with PA-TACE.

**Method:**

We enrolled 112 patients (75 in the training cohort and 37 in the validation cohort) with HCC treated with PA-TACE after surgical resection at the Affiliated Hospital of Nantong University between January 2012 and June 2015. The independent OS predictors were determined using univariate and multivariate regression analyses. Decision curve analyses and time-dependent receiver operating characteristic curve analysis was used to verify the prognostic performance of the different models; the best model was selected to establish a multi-dimensional nomogram for predicting the OS of HCC patients treated with PA-TACE.

**Result:**

Multivariate regression analyses indicated that rim-like arterial phase enhancement (IRE), peritumor capsule (PTC), and alanine aminotransferase to hemoglobin ratio (AHR) were independent predictors of OS after PA-TACE. The combination of AHR had the best clinical net benefit and we constructed a prognostic nomogram based on IRE, PTC, and AHR. The calibration curve showed good fit between the predicted nomogram’s curve and the observed curve.

**Conclusion:**

Our preliminary study confirmed the prognostic value of AHR, PTC, and IRE and established a nomogram that can predict the OS after PA-TACE treatment in patients with HCC.

## Introduction

Hepatocellular carcinoma (HCC) is one of the most common malignant tumors, the fifth most common malignancy, and the third-leading cause of cancer-related death worldwide; moreover, its incidence and mortality rates are increasing (1). Owing to its high recurrence rate, long-term effects of surgical resection are poor (2, 3). Therefore, transarterial chemoembolization (TACE) and other treatments after surgical resection are increasingly accepted by clinicians (4).

Simultaneously killing cells by restricting blood supply and infusion with chemotherapy drugs are the main contributions of TACE to treating HCC (5). The latest Chinese ***Guidelines for Diagnosis and Treatment of Primary Liver Cancer (2022 Edition)*** recommends postoperative adjuvant TACE (PA-TACE) in case of high-risk recurrence factors, such as tumor thrombus formation and multiple tumors, to reduce recurrence and prolong survival. PA-TACE has been shown to improve the overall survival (OS) of patients with HCC and portal vein tumor thrombus after surgical resection or patients diagnosed with B stage tumors according to the Barcelona Clinic Liver Cancer evaluation system (6, 7). However, whether patients can benefit from PA-TACE remains controversial (8), As the responses to TACE in patients with HCC is variable (9), it is necessary to identify the patients who can benefit from PA-TACE and implement individualized treatments.

The prognosis of HCC is closely related to liver function and systemic conditions. Alanine aminotransferase (ALT) and aspartate aminotransferase (AST) are often indicators of liver function in clinical settings. Many studies have reported the relationship of ALT with the recurrence and low survival rate of hepatitis B virus-related HCC (10, 11). Hemoglobin (Hb) can reflect anemia and be used to predict HCC’s prognosis (12). The alanine aminotransferase to hemoglobin ratio (AHR) has been reported to predict progression-free survival in patients treated with TACE (13). However, the relationship between AHR and OS after TACE remains unclear.

In addition, the tumor nature is itself an important factor affecting the prognosis of HCC (14). Imaging is an important examination method for identifying tumor nature before surgery. Computed tomography (CT) is the preferred examination method for HCC because of its efficiency and economic advantages (15). CT is often used to predict the prognosis of TACE, but its radiological features are neglected in this case. However, the predictive ability of multiple markers is often more advantageous than using a single marker. Therefore, in the prognostic model constructed in this study, we included two additional radiological features (rim-like arterial phase enhancement [IRE] and peritumor capsule [PTC]).

This study aimed to explore the relationship between the radiological features of HCC, AHR after surgical resection, and prognosis of PA-TACE. We also constructed a prognostic nomogram including IRE, PTC, and AHR to predict the OS of patients undergoing PA-TACE.

## Materials and methods

### Study patients

Between January 2012 and June 2015, we identified a consecutive series of 149 patients with HCC who underwent PA-TACE at the Affiliated Hospital of Nantong University, Nantong, China. The inclusion criteria for this study were: (1) clinical diagnosis of HCC; (2) PA-TACE within 2 months after surgical resection; (3) complete preoperative images and postoperative serum data; (4) no other malignant tumors; and (5) no extrahepatic metastasis. Thirty-seven patients were excluded based on these criteria: (1) receiving other therapies before surgical resection (n = 24); (2) having radiological images from other hospitals (n = 10); and (3) missing follow-up data (n = 3). Ultimately, 112 patients were included in the study. All patients provided written informed consent before surgery. The patients were randomly divided into training (n = 75) and validation (n = 37) cohorts.

### Data collection and follow-up

The following clinical and laboratory data were extracted from the medical records system: name; age; sex; presence of cirrhosis; alpha-fetoprotein, AST, ALT, Hb, and albumin levels; white cell, lymphocyte, and platelet counts; and AHR, defined as the ratio of ALT to Hb at the first laboratory examination after surgical resection. Cut-off AHR values were determined using receiver operating characteristic (ROC) curve analysis.

**Figure 1:**
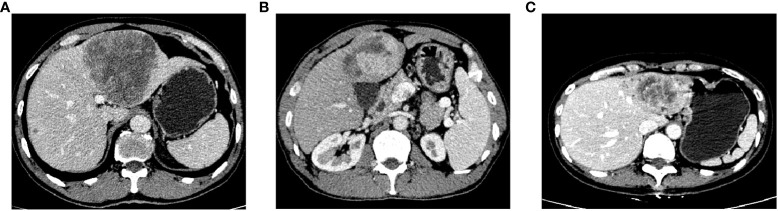
Enhanced CT for PTC **(A)**, IRE **(B)**, and both PTC and IRE **(C)**, CT, computed tomography; PTC, peritumor capsule; IRE, rim-like arterial phase enhancement.

IRE was defined as irregular hyper-enhancement at the tumor edge and hypo-enhancement at the center in the arterial phase. PTC was defined as a capsule-like lesion with clear boundary hyper-enhancement around the tumor parenchyma in the arterial phase (**Figures 1A–C**). Two radiologists with >5 years of work experience judged whether the patient had IRE or PTC, while being blinded to the patients’ AHR. OS was defined as the time from PA-TACE to death or the last follow-up.

### Construction of models and the nomogram

We developed two models to compare the training and validation cohorts. The prognostic value of the two models was determined by decision curve analyses (DCA) and time-dependent ROC analyses. The best model was selected to construct the nomogram for the entire cohort. Model 1 included IRE, PTC, and AHR, and Model 2 included only IRE and PTC.

### Statistical analysis

The t-test was used to analyze continuous variables with a normal distribution. The Wilcoxon rank-sum test was used to analyze continuous variables not conforming to a normal distribution. For classified data, we chose the χ^2^ test when each level met the requirements of frequency >5 and total sample size >40; otherwise, the Fisher precision probability test was used. We used the “survival” package in R software, version 3.6.3 (R Foundation for Statistical Computing, Vienna, Austria), to perform univariate and multivariate Cox regression analyses and used variables with a *P*-value <0.1 in univariate analysis for multivariate analysis. In addition, we used the “survival” and “stdca.R” packages in R to perform DCA for evaluating the clinical application of different models and the nomogram (16). Time-dependent ROC curves were analyzed using the “timeROC” package in R and visualized by the ggplot2 package (17). The nomogram and calibration curves were analyzed and visualized using the “rms” package in R.

All statistical analyses were performed using SPSS, version 26.0 (IBM Corp., Armonk, NY, USA) and R software version 3.6.3. We considered a *P*-value <0.05 as statistically significant.

## Results

### Patient characteristics

The patient characteristics of the two cohorts are shown in **Table 1**. The death status (*P* = 0.139) and OS (*P* = 0.194) did not significantly differ between the training and validation cohorts. In addition, the clinical parameters, laboratory data, and radiological characteristics were similar between the training and validation cohorts. These findings indicate no significant difference in baseline data between the two cohorts; the two cohorts were homogeneous and comparable, which served as basis for our subsequent analysis.

**Table 1:** Baseline patient data in the two cohorts.

Characteristic	Training cohort n = 75	Validation cohort n = 37	*P*
**Sex, n (%)**			0.259
**Female**	12 (16%)	10 (27%)	
**Male**	63 (84%)	27 (73%)	
**AFP, n (%)**			0.211
**<400**	53 (70.7%)	21 (56.8%)	
**≥400**	22 (29.3%)	16 (43.2%)	
**IRE, n (%)**			0.081
**Absent**	47 (62.7%)	16 (43.2%)	
**Present**	28 (37.3%)	21 (56.8%)	
**PTC, n (%)**			1.000
**Absent**	42 (56%)	21 (56.8%)	
**Present**	33 (44%)	16 (43.2%)	
**Child-pugh, n (%)**			1.000
**A**	3 (4%)	1 (2.7%)	
**B**	72 (96%)	36 (97.3%)	
**Cirrhosis, n (%)**			0.794
**Absent**	23 (30.7%)	13 (35.1%)	
**Present**	52 (69.3%)	24 (64.9%)	
**Status, n (%)**			0.139
**Alive**	47 (62.7%)	17 (45.9%)	
**Dead**	28 (37.3%)	20 (54.1%)	
**Age, mean ± SD**	55.17 ± 9.66	54.49 ± 9.39	0.722
**AST, mean ± SD**	41.77 ± 33.25	39.32 ± 14.19	0.669
**ALT, mean ± SD**	45.72 ± 39.78	41 ± 24.38	0.509
**ALB, mean ± SD**	38.58 ± 4.58	37.6 ± 4.05	0.269
**WBC, mean ± SD**	5.09 ± 1.5	4.72 ± 1.1	0.193
**LY, mean ± SD**	1.65 ± 0.53	1.51 ± 0.46	0.160
**PLT, mean ± SD**	153.31 ± 61.86	151.46 ± 57.89	0.880
**Hb, mean ± SD**	142.59 ± 16.37	137.32 ± 13.87	0.096
**OS day, mean ± SD**	1666.93 ± 800.56	1459.05 ± 773.56	0.194

AFP, alpha-fetoprotein; IRE, irregular rim-like arterial phase enhancement; PTC, peritumor capsule; AST, aspartate aminotransferase; ALT, alanine aminotransferase; ALB, albumin; WBC, white blood cell; LY, lymphocyte count; PLT, platelet count; Hb, hemoglobin; OS, overall survival; SD, standard deviation.

### Prognostic factors of OS in the training cohort

First, we determined the optimal AHR cut-off value to be 0.940, with an area under the curve (AUC) of 0.554 (95% confidence interval [CI]: 0.410–0.698), sensitivity of 28.6%, and specificity of 93.6% (**Table 2**). Univariate analysis showed that PTC (hazard ratio [HR] = 0.314; 95% CI: 0.135–0.732; *P* = 0.007) and AHR >0.94 (HR = 6.376; 95% CI: 2.584–15.735; *P <*0.001) were risk factors for OS (**Table 3**). Second, we performed multivariate analysis on parameters with a *P*-value <0.1 in univariate analysis. The result showed that IRE (HR = 2.8; 95% CI: 1.186–6.608; *P* = 0.019), PTC (HR = 0.401; 95% CI: 0.166–0.965; *P* = 0.041), and AHR >0.94 (HR = 6.698; 95% CI: 2.561–17.519; *P <*0.001) were independent predictors of OS (**Table 3**). We observed no significant differences in IRE in univariate analysis (hazard ratio [HR] = 2.204; 95% CI: 0.990–4.906; *P* = 0.053). However, after multivariate analysis, IRE became an independent prognostic factor for OS.

**Table 2:** \Cut-off value and AUC of the AHR after surgical resection.

Parameter	Cut-off value	AUC	Sensitivity (%)	Specificity (%)	95% CI of AUC
AHR	0.940	0.554	28.6%	93.6%	0.410-0.698

AUC, area under the curve; CI, confidence interval; AHR, alanine aminotransferase to hemoglobin ratio.

**Table 3:** Univariable and multivariable Cox analyses of OS in the training cohort.

Characteristics	Total (N)	HR (95% CI) Univariate analysis	*P*	HR (95% CI) Multivariate analysis	*P*
**Age**	75	1.024 (0.983–1.066)	0.252		
**Child-pugh**	75		0.783		
**B**	72	Reference			
**A**	3	1.329 (0.176–10.038)	0.783		
**Cirrhosis**	75	1.128 (0.501–2.539)	0.772		
**AFP**	75		0.114		
**<400**	53	Reference			
**≥400**	22	1.917 (0.855–4.300)	0.114		
**IRE**	75		0.053		
**Absent**	47	Reference			
**Present**	28	2.204 (0.990–4.906)	0.053	2.800 (1.186–6.608)	0.019
**PTC**	75		0.007		
**Absent**	42	Reference			
**Present**	33	0.314 (0.135–0.732)	0.007	0.401 (0.166–0.965)	0.041
**ALT**	75	1.000 (0.999–1.001)	0.841		
**Hb**	75	1.009 (0.988–1.030)	0.422		
**AHR**	75		< 0.001		
**≤ 0.94**	64	Reference			
**> 0.94**	11	6.376 (2.584–15.735)	< 0.001	6.698 (2.561–17.519)	< 0.001

HR, hazard ratio; OS, overall survival; AFP, alpha-fetoprotein; IRE, irregular rim-like arterial phase enhancement; PTC, peritumor capsule; ALT, alanine aminotransferase; OS, overall survival; SD, standard deviation; AHR, alanine aminotransferase to hemoglobin ratio.

### Optimal model for predicting the OS of patients treated with PA-TACE

**Figure 2:**
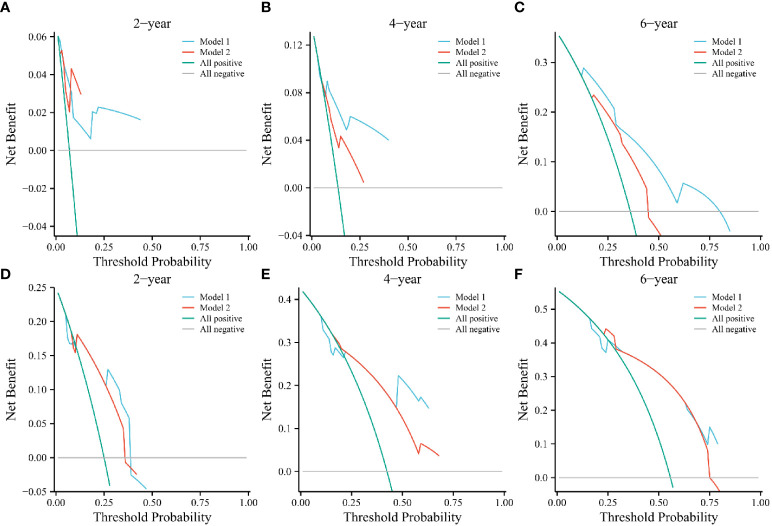
Decision curve analyses of Models 1 and 2 at 2, 4, and 6 years in the training **(A–C)** and validation cohorts **(D–F)**. The green and gray line indicates that all patients were dead or alive, respectively. The blue and red line indicates the clinical net benefit of Model 1 and 2 at different threshold probabilities.

We established two models: Model 1, consisting of IRE, PTC, and the AHR; and Model 2, consisting of IRE and PTC. This design allowed us to observe differences in radiological characteristics with or without the AHR. Based on the 2-year DCA of the training cohort, Models 1 and 2 had a similar clinical net benefit (**Figure 2A**), but the clinical net benefit of Model 1 at 4 and 6 years was better than that of Model 2 in the training cohort (**Figures 2A–C**). In the validation cohort, we found that the net clinical benefit of Model 1 at 2, 4, and 6 years was better than that of Model 2 (**Figures 2D–F**).

**Figure 3:**
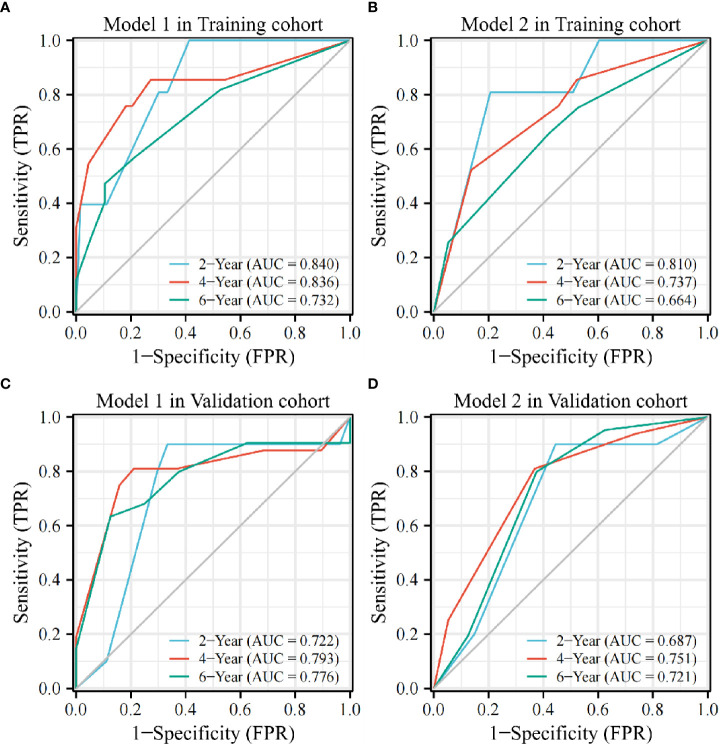
The AUC value of Model 1 is higher than that of Model 2 at 2, 4, and 6 years in the training **(A, B)** and validation cohorts **(C, D)**.AUC, area under the curve; TPR, true positive rate; FPR, false positive rate.

The time-dependent ROC curves indicated AUC values of Model 1 in the training cohort of 0.840, 0.836, and 0.732 at 2, 4, and 6 years, respectively. In contrast, those in the validation cohort were 0.722, 0.793, and 0.776 at 2, 4, and 6 years, respectively (**Figures 3A, C**). However, the AUC values of Model 2 in the training cohort were 0.810, 0.737, and 0.664 at 2, 4, and 6 years, respectively, whereas those in the validation cohort were 0.687, 0.751, and 0.721 at 2, 4, and 6 years, respectively; lower than those of Model 1 (**Figures 3B, D**).

The results above show better predictive performance of Model 1 compared to Model 2. Based on its performance, we selected Model 1 as the final model.

### Establishment and verification of the nomogram

**Figure 4:**
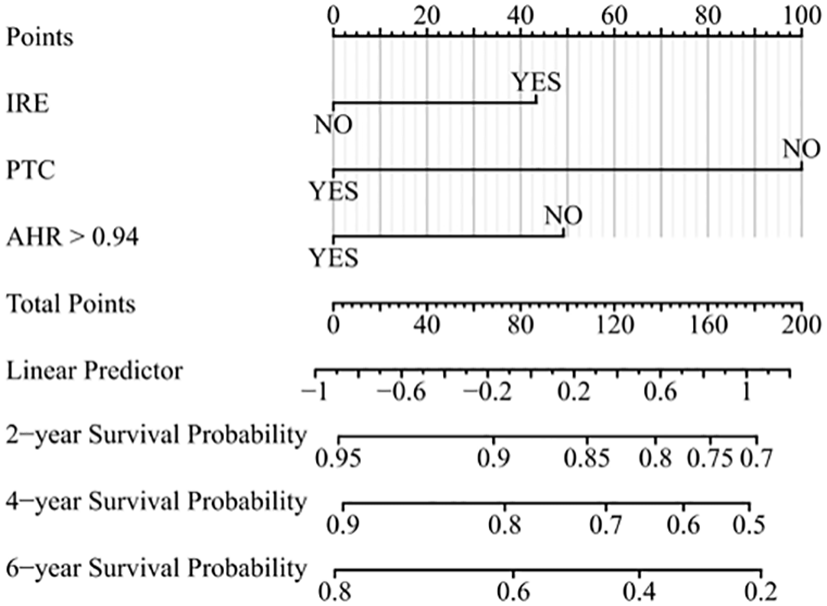
Prognostic nomogram for predicting OS of patients treated with PA-TACE. OS, overall survival; PA-TACE, postoperative adjuvant transarterial chemoembolization; IRE, rim-like arterial phase enhancement; PTC, peritumor capsule; AHR, alanine aminotransferase to hemoglobin ratio.

**Figure 5:**
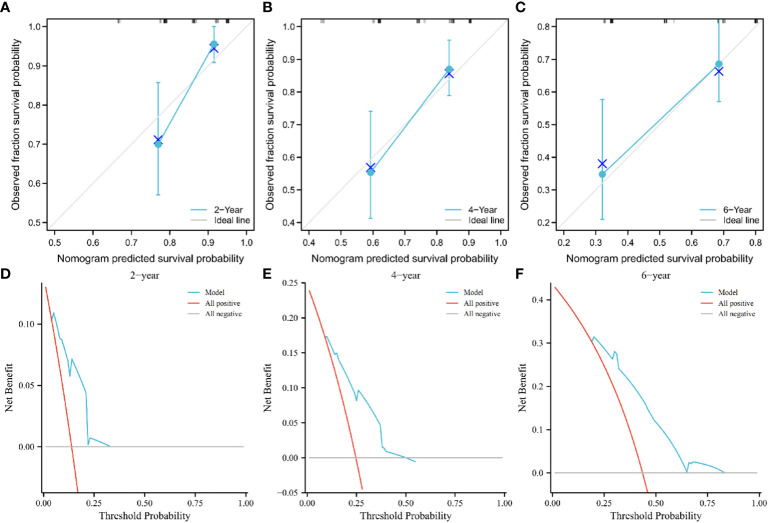
Calibration curve showing good fit between the predicted nomogram curve and the observed curve **(A–C)**. DCA of the nomogram at 2, 4, and 6 years. The green and gray line indicates that all patients were dead or alive, respectively. The red line indicates the clinical net benefit of the nomogram at different threshold probabilities **(D–F)**. DCA, decision curve analysis.

We found superior predictive ability of the combination of three indicators to the individual predictive ability. Further, we used PTC, IRE, and the AHR to establish a nomogram to predict the OS of patients treated with PA-TACE (**Figure 4**). The calibration curve showed a good fit between the predicted curve of the nomogram and the observed curve at 2, 4, and 6 years (**Figures 5A–C**). Moreover, DCA showed that the nomogram had an excellent net clinical benefit at 2, 4, and 6 years (**Figures 5D–F**).

## Discussion

In this study, we constructed two models to predict PA-TACE prognosis. Using DCA and ROC analysis, we found better predictive performance of Model 1 compared to Model 2. This shows better predictive performance of the multi-dimensional model than that of simple radiological features. We used these three indicators to establish a nomogram to predict the OS after PA-TACE. The calibration curve showed a good fit between the predicted and observed curves of the nomogram. Clinically, ALT and Hb are part of routine blood tests on admission or discharge. In addition, an important means for diagnosing HCC is enhanced CT examination. The enhanced CT image allows identifying whether the patient has PTC or IRE, information usually included in the radiologist’s report. Intervening doctors can quickly obtain these three indicators and predict the OS of patients using our nomogram for assistive judgment of patient suitability for PA-TACE. The current scoring system for TACE includes patients who only received TACE treatment. The data in this study included patients who received PA-TACE after surgery; a more specific concept. Compared with other scoring systems such as HAP and ART, the data in our prognostic model is easier to obtain, having verified clinical effects (18–20).

Increased ALT indicates damage to liver function and represents the formation of a tumor microenvironment conducive to the development of HCC (21, 22). A decrease in Hb indicates that the oxygen carried by red blood cells is decreased and that the tissue is in a state of hypoxia. A hypoxic microenvironment may increase the expression of angiogenic factors, such as hypoxia-inducible factor 1α and vascular endothelial growth factor, associated with poor prognosis of TACE (23–25). In this study, we found that ALT and Hb cannot be used to predict the OS after PA-TACE, but the AHR was an independent prognostic factor, which may result from the interaction of many factors. Thus, unveiling the underlying mechanism requires further research.

The impact of the presence or absence of PTC on prognosis is controversial (26). Some studies have suggested that PTC prevents HCC invasion. In clinical practice, presence or absence of PTC affects the surgical resection method. The scope of HCC resection with a capsule is often smaller, resulting in less damage to liver function. HCC without a capsule is more likely to spread and cause more significant damage to liver function after surgical resection, which may lead to a worse clinical response of patients to PA-TACE. Surgeons often worry about HCC invasion depth without a capsule and tend to expand the resection area (26–28). In our study, patients with PTC had a better prognosis after PA-TACE than those without. In previous studies, IRE was considered an invasive marker of HCC and associated with early recurrence after radiofrequency ablation (29). Similarly, the present study also found that patients with IRE had a worse prognosis than those without.

This study has few limitations. In the past, PA-TACE was not often used after surgical resection; therefore, few patients were enrolled. Despite the relatively small sample size of our study, our predictive model was verified in the validation cohort. Moreover, our study is a single-center study. In the future, to improve our prognosis model, we will cooperate with other hospitals to improve on the generalizability of our nomogram.

In conclusion, our study used radiological features and serum indicators to establish a nomogram for predicting the OS of patients with HCC treated with PA-TACE. This predictive model can quickly determine whether patients can benefit from PA-TACE after surgical resection of HCC.

## Data availability statement

The raw data supporting the conclusions of this article will be made available by the authors, without undue reservation.

## Ethics statement

Written informed consent was obtained from the individual(s) for the publication of any potentially identifiable images or data included in this article.

## Author contributions

ZX and YZ contributed equally to this research.WZ, AS, and HZ contributed to research concept and design. ZX and YZ contributed to data analysis and interpretation and manuscript writing and editing. WL and LW contributed to acquisition of image. JiaZ, CL, KC, JY, and JiaZ contributed to data acquisition. All authors contributed to the research and agreed to be accountable for the content of the work.

## Funding

This research was supported by grants from the Graduate Innovation Program of Jiangsu Province (No. SJCX21-1466, No.SJCX22-1636, and No.SJCX22-1634), National Natural Science Foundation of China (grant: 82102784), and Natural Science Foundation of Jiangsu Province (BK20221275).

## Acknowledgments

We would like to thank Editage (www.editage.cn) for English language editing.

## Conflict of interest

The authors declare that the research was conducted in the absence of any commercial or financial relationships that could be construed as a potential conflict of interest.

## Publisher’s note

All claims expressed in this article are solely those of the authors and do not necessarily represent those of their affiliated organizations, or those of the publisher, the editors and the reviewers. Any product that may be evaluated in this article, or claim that may be made by its manufacturer, is not guaranteed or endorsed by the publisher.

## References

[1] Calderaro J Seraphin TP Luedde T Simon TG. Artificial intelligence for the prevention and clinical management of hepatocellular carcinoma. J Hepatol (2022) 76(6):1348–61. doi: 10.1016/j.jhep.2022.01.014 PMC9126418 35589255

[2] Tabrizian P Jibara G Shrager B Schwartz M Roayaie S. Recurrence of hepatocellular cancer after resection: patterns, treatments, and prognosis. Ann Surg (2015) 261(5):947–55. doi: 10.1097/SLA.0000000000000710 25010665

[3] Mao S Yu X Sun J Yang Y Shan Y Sun J. Development of nomogram models of inflammatory markers based on clinical database to predict prognosis for hepatocellular carcinoma after surgical resection. BMC Cancer (2022) 22(1):249. doi: 10.1186/s12885-022-09345-2 35255845 PMC8900373

[4] Wang PX Sun YF Zhou KQ Cheng JW Hu B Guo W. Circulating tumor cells are an indicator for the administration of adjuvant transarterial chemoembolization in hepatocellular carcinoma: A single-center, retrospective, propensity-matched study. Clin Transl Med (2020) 10(3):e137. doi: 10.1002/ctm2.137 32702202 PMC7418815

[5] Li QJ He MK Chen HW Fang WQ Zhou YM Xu L. Hepatic arterial infusion of oxaliplatin, fluorouracil, and leucovorin versus transarterial chemoembolization for Large hepatocellular carcinoma: A randomized phase III trial. J Clin Oncol (2022) 40(2):150–60. doi: 10.1200/JCO.21.00608 34648352

[6] Hu S Gan W Qiao L Ye C Wu D Liao B. A new prognostic algorithm predicting HCC recurrence in patients with Barcelona clinic liver cancer stage b who received PA-TACE. Front Oncol (2021) 11:742630. doi: 10.3389/fonc.2021.742630 34745962 PMC8566809

[7] Liu F Guo X Dong W Zhang W Wei S Zhang S. Postoperative adjuvant TACE-associated nomogram for predicting the prognosis of resectable hepatocellular carcinoma with portal vein tumor thrombus after liver resection. Int J Biol Sci (2020) 16(16):3210–20. doi: 10.7150/ijbs.46896 PMC7645989 33162826

[8] Tong Y Li Z Liang Y Yu H Liang X Liu H. Postoperative adjuvant TACE for patients of hepatocellular carcinoma in AJCC stage I: friend or foe? a propensity score analysis. Oncotarget (2017) 8(16):26671–8. doi: 10.18632/oncotarget.15793 PMC5432288 28460456

[9] Tang Y Wu Y Xue M Zhu B Fan W Li J. A 10-gene signature identified by machine learning for predicting the response to transarterial chemoembolization in patients with hepatocellular carcinoma. J Oncol (2022) (2022) 3822773. doi: 10.1155/2022/3822773 35111225 PMC8803430

[10] Cheung YS Chan HL Wong J Lee KF Poon TC Wong N. Elevated perioperative transaminase level predicts intrahepatic recurrence in hepatitis b-related hepatocellular carcinoma after curative hepatectomy. Asian J Surg (2008) 31(2):41–9. doi: 10.1016/S1015-9584(08)60056-1 18490213

[11] Tarao K Takemiya S Tamai S Sugimasa Y Ohkawa S Akaike M. Relationship between the recurrence of hepatocellular carcinoma (HCC) and serum alanine aminotransferase levels in hepatectomized patients with hepatitis c virus-associated cirrhosis and HCC. Cancer (1997) 79(4):688–94. doi: 10.1002/(SICI)1097-0142(19970215)79:4<688::AID-CNCR5>3.0.CO;2-A 9024706

[12] Finkelmeier F Bettinger D Köberle V Schultheiß M Zeuzem S Kronenberger B. Single measurement of hemoglobin predicts outcome of HCC patients. Med Oncol (2014) 31(1):806. doi: 10.1007/s12032-013-0806-2 24326985

[13] Lin ZH Li X Hong YF Ma XK Wu DH Huang M. Alanine aminotransferase to hemoglobin ratio is an indicator for disease progression for hepatocellular carcinoma patients receiving transcatheter arterial chemoembolization. Tumour Biol (2016) 37(3):2951–9. doi: 10.1007/s13277-015-4082-y 26411670

[14] Yoneda N Matsui O Kobayashi S Kitao A Kozaka K Inoue D. : Current status of imaging biomarkers predicting the biological nature of hepatocellular carcinoma. Jpn J Radiol (2019) 37(3):191–208. doi: 10.1007/s11604-019-00817-3 30712167

[15] Chen X Yang Z Deng J. Use of 64-slice spiral CT examinations for hepatocellular carcinoma (DR LU). J buon (2019) 24(4):1435–40. 31646788

[16] Vickers AJ Elkin EB. Decision curve analysis: a novel method for evaluating prediction models. Med Decis Making (2006) 26(6):565–74. doi: 10.1177/0272989X06295361 PMC2577036 17099194

[17] Ginestet C. ggplot2: Elegant Graphics for Data Analysis. . Journal of the Royal Statistical Society (2011) 174(1):245–246. doi: 10.1111/j.1467-985X.2010.00676_9

[18] Sieghart W Hucke F Pinter M Graziadei I Vogel W Müller C. The ART of decision making: retreatment with transarterial chemoembolization in patients with hepatocellular carcinoma. Hepatology (2013) 57(6):2261–73. doi: 10.1002/hep.26256 23316013

[19] Kadalayil L Benini R Pallan L O'Beirne J Marelli L Yu D. A simple prognostic scoring system for patients receiving transarterial embolisation for hepatocellular cancer. Ann Oncol (2013) 24(10):2565–70. doi: 10.1093/annonc/mdt247 PMC4023407 23857958

[20] Hucke F Sieghart W Pinter M Graziadei I Vogel W Müller C. The ART-strategy: sequential assessment of the ART score predicts outcome of patients with hepatocellular carcinoma re-treated with TACE. J Hepatol (2014) 60(1):118–26. doi: 10.1016/j.jhep.2013.08.022 24012941

[21] Başar O Yimaz B Ekiz F Giniş Z Altinbaş A Aktaş B. Non-invasive tests in prediction of liver fibrosis in chronic hepatitis b and comparison with post-antiviral treatment results. Clin Res Hepatol Gastroenterol (2013) 37(2):152–8. doi: 10.1016/j.clinre.2012.07.003 23391746

[22] Suh SW Lee JM You T Choi YR Yi NJ Lee KW. Hepatic venous congestion in living donor grafts in liver transplantation: is there an effect on hepatocellular carcinoma recurrence? Liver Transpl (2014) 20(7):784–90. doi: 10.1002/lt.23877 24668935

[23] Lin ZH Jiang JR Ma XK Chen J Li HP Li X. Prognostic value of serum HIF-1α change following transarterial chemoembolization in hepatocellular carcinoma. Clin Exp Med (2021) 21(1):109–20. doi: 10.1007/s10238-020-00667-8 33037574

[24] Huang M Wang L Chen J Bai M Zhou C Liu S. Regulation of COX-2 expression and epithelial-to-mesenchymal transition by hypoxia-inducible factor-1α is associated with poor prognosis in hepatocellular carcinoma patients post TACE surgery. Int J Oncol (2016) 48(5):2144–54. doi: 10.3892/ijo.2016.3421 PMC4809660 26984380

[25] Liu K Min XL Peng J Yang K Yang L Zhang XM. The changes of HIF-1α and VEGF expression after TACE in patients with hepatocellular carcinoma. J Clin Med Res (2016) 8(4):297–302. doi: 10.14740/jocmr2496w 26985249 PMC4780492

[26] Kim BK Kim KA An C Yoo EJ Park JY Kim DY. Prognostic role of magnetic resonance imaging vs. computed tomography for hepatocellular carcinoma undergoing chemoembolization. Liver Int (2015) 35(6):1722–30. doi: 10.1111/liv.12751 25444138

[27] Song L Li J Luo Y. The importance of a nonsmooth tumor margin and incomplete tumor capsule in predicting HCC microvascular invasion on preoperative imaging examination: a systematic review and meta-analysis. Clin Imaging (2021) 76:77–82. doi: 10.1016/j.clinimag.2020.11.057 33578134

[28] Zhu YJ Feng B Wang BZ Wang S Ye F Ma XH. [Value of gadolinium ethoxybenzyl diethylenetriamine pentaacetic acid enhanced magnetic resonance imaging and diffusion-weighted MR imaging in predicting microvascular invasion in hepatocellular carcinoma and the prognostic significance]. Zhonghua zhong liu za zhi. (2021) 43(3):312–7. doi: 10.3760/cma.j.cn112152-20191009-00652 33752311

[29] Kang TW Rhim H Lee J Song KD Lee MW Kim YS. Magnetic resonance imaging with gadoxetic acid for local tumour progression after radiofrequency ablation in patients with hepatocellular carcinoma. Eur Radiol (2016) 26(10):3437–46. doi: 10.1007/s00330-015-4190-5 26747262

